# Identification potential biomarkers in pulmonary tuberculosis and latent infection based on bioinformatics analysis

**DOI:** 10.1186/s12879-016-1822-6

**Published:** 2016-09-21

**Authors:** Xue-Bing Qin, Wei-Jue Zhang, Lin Zou, Pei-Jia Huang, Bao-Jun Sun

**Affiliations:** 1 Nanlou Respiratory Diseases Department, Chinese PLA General Hospital, No. 28, Fuxing Road, Beijing, 100853 China; 2 Department of Respiratory, Chinese PLA General Hospital, Beijing, 100853 China; 3 Medical College, Nankai University, Tianjin, 300071 China; 4 Nanlou Health Care Department, Chinese PLA General Hospital, Beijing, 100853 China

**Keywords:** Pulmonary tuberculosis, *Mycobacterium tuberculosis*, Bioinformatics analysis, Differentially expressed genes, Biomarker

## Abstract

**Background:**

The study aimed to identify the potential biomarkers in pulmonary tuberculosis (TB) and TB latent infection based on bioinformatics analysis.

**Methods:**

The microarray data of GSE57736 were downloaded from Gene Expression Omnibus database. A total of 7 pulmonary TB and 8 latent infection samples were used to identify the differentially expressed genes (DEGs). The protein-protein interaction (PPI) network was constructed by Cytoscape software. Then network-based neighborhood scoring analysis was performed to identify the important genes. Furthermore, the functional enrichment analysis, correlation analysis and logistic regression analysis for the identified important genes were performed.

**Results:**

A total of 1084 DEGs were identified, including 565 down- and 519 up-regulated genes. The PPI network was constructed with 446 nodes and 768 edges. Down-regulated genes RIC8 guanine nucleotide exchange factor A (*RIC8A*), basic leucine zipper transcription factor, ATF-like (*BATF*) and microtubule associated monooxygenase, calponin LIM domain containing 1 (*MICAL1*) and up-regulated genes ATPase, Na+/K+ transporting, alpha 4 polypeptide (*ATP1A4*), histone cluster 1, H3c (*HIST1H3C*), histone cluster 2, H3d (*HIST2H3D*), histone cluster 1, H3e (*HIST1H3E*) and tyrosine kinase 2 (*TYK2*) were selected as important genes in network-based neighborhood scoring analysis. The functional enrichment analysis results showed that these important DEGs were mainly enriched in regulation of osteoblast differentiation and nucleoside triphosphate biosynthetic process. The gene pairs *RIC8A*-*ATP1A4*, *HIST1H3C*-*HIST2H3D*, *HIST1H3E*-*BATF* and *MICAL1*-*TYK2* were identified with high positive correlations. Besides, these genes were selected as significant feature genes in logistic regression analysis.

**Conclusions:**

The genes such as *RIC8A*, *ATP1A4*, *HIST1H3C*, *HIST2H3D*, *HIST1H3E*, *BATF*, *MICAL1* and *TYK2* may be potential biomarkers in pulmonary TB or TB latent infection.

## Background

Pulmonary tuberculosis (TB) is a widespread and fatal infectious disease. It is caused by various strains of mycobacteria, usually *Mycobacterium tuberculosis* [1]. It is estimated that one third of the world’s population are infected with *M. tuberculosis* [2]. More than 90 % of infected individuals remain asymptomatic with a latent infection [3]. With aging or immune system deteriorating, *M. tuberculosis* can reactivate and cause severe pulmonary TB [4]. Roughly 10 % of the latent infections can progress to active TB. The general signs and symptoms of this disease include fever, chills, night sweats, loss of appetite, weight loss, and fatigue [5]. Approximately, there are 9 million newly diagnosed cases of pulmonary TB and 1.5 million deaths annually, mostly in developing countries [6]. Therefore, uncovering therapeutic biomarkers in pulmonary TB would supply new insights for the diagnosis and treatment of this disease.

Numerous studies have been done to investigate the potential biomarkers for the treatment of pulmonary TB. For example, the serum CA-125 level is found significantly higher in active pulmonary TB than in inactive TB or normal sample, suggesting that CA-125 may be a beneficial parameter in determination of pulmonary TB activity [7]. Pollock et al. [8] suggested that *M. tuberculosis* Rv1681 protein was a diagnostic marker of active pulmonary TB. Additionally, Chowdhury et al. [9] reported that the serum interleukin (IL)-6 level of the active pulmonary TB patients following anti-tuberculosis drug therapy played an important role in immune-protection of the host against *M. tuberculosis* infection. Although many factors have been found, the diagnostic efficiency of pulmonary TB is still unsatisfactory [10]. Therefore, it is necessary to identify novel potential therapeutic biomarkers in pulmonary TB.

In the present study, the microarrays data GSE57736 were downloaded to identify the differentially expressed genes (DEGs) between pulmonary TB and latent tuberculosis infection samples. This dataset is deposited by Guerra-Laso et al. [11], the study of whom demonstrates that IL-26 is a candidate gene for TB susceptibility. In this study, we aimed to use different bioinformatics method to identify the DEGs between the two kinds of samples. Based on the obtained DEGs, we performed protein-protein interaction (PPI) network construction and network-based neighborhood scoring analysis. Besides, the hierarchical clustering analysis, functional enrichment analysis, correlation analysis and logistic regression analysis of DEGs were performed as well. Findings of this study may help to explore potential targets for the diagnosis and treatment in pulmonary TB.

### Methods

#### Affymetrix microarray data

The array data of GSE57736 based on the platform of GPL13497 (Agilent-026652 Whole Human Genome Microarray 4x44K v2) was downloaded from Gene Expression Omnibus database, which was deposited by Guerra-Laso *et al.* [11]. The dataset available in this analysis contained 15 peripheral blood samples from seven pulmonary TB patients and eight latent tuberculosis infections. Among the seven pulmonary TB patients, there were three men and four women (average 82.7 years) with different clinical conditions: psoriasis (one patient), previous heart failure (one patient), arterial hypertension (two patients), bronchial asthma (one patient), chronic obstructive pulmonary disease (two patients), and prostate cancer (one patient). The eight latent tuberculosis infection samples included six men and two women (average 81.1 years), which had scored a positive result in the QuantiFERON-TB Gold in-tube test (Cellestis, Carnegie, Vic., Australia).

#### Data preprocessing and differential expression analysis

The probe IDs were converted into corresponding gene symbols based on the annotation information on the platform. When multiple probes corresponded to a same gene, the average expression value was calculated to represent the gene expression level. The limma package [12] in R was used to identify DEGs between pulmonary TB and TB latent infection samples. The Benjamin and Hochberg (BH) [13] method was used to adjust the raw *p*-values [false discovery rate (FDR)]. Then, log_2_-fold change (log_2_FC) was calculated. Only genes with |log_2_FC| > 1.0 and FDR < 0.05 were selected as DEGs.

#### PPI network construction

Human Protein Reference Database (HPRD, http://www.hprd.org/) [14] is a database of curated proteomic information pertaining to human proteins. Search Tool for the Retrieval of Interacting Genes (STRING, http://string.embl.de/) [15] is an online database which collects comprehensive information of proteins. In our study, the DEGs were mapped into STRING and HPRD databases to identify significant protein pairs with confidence score > 0.4. Then the PPI network was constructed based on these protein pairs using Cytoscape software [16].

#### Network-based neighborhood scoring

Neighborhood scoring [17] is a local method for prioritizing candidates based on the distribution of DEGs in the network. Gene in PPI network was assigned a score, which was based on its FC and the FC of its neighbors. The score of each node in PPI network was calculated with the neighborhood scoring method [18]. When the hub node and its neighborhood nodes were significantly highly expressed, the score > 0; When the hub node and its neighborhood nodes were significantly lowly expressed, the score < 0. Therefore, the top 50 nodes with higher scores and the last 50 nodes with lower scores were identified as important genes.

In order to confirm the efficiency of these important genes differentiating pulmonary TB and TB latent infection samples, hierarchical clustering analysis of the important genes was performed using cluster software [19]. The results were presented by TreeView software [20]. The expression profile data were filtered and normalized using cluster software. In detail, genes that were expressed in at least 80 % of the samples were selected. Besides, the genes and samples were normalized with median center method [21].

#### Functional enrichment analysis

Gene Ontology (GO, http://www.geneontology.org) database [22] is a collection of a large number of gene annotation terms. Kyoto Encyclopedia of Genes and Genomes (KEGG, http://www.genome.ad.jp/kegg/) knowledge database [23] is applied to identify the functional and metabolic pathway. Database for Annotation, Visualization and Integrated Discovery (DAVID, https://david.ncifcrf.gov/) [24] is a tool that provides a comprehensive set of functional annotation for large list of genes. In this study, the important genes were performed GO and KEGG pathway enrichment analyses with DAVID. With the enrichment threshold of *p*-value < 0.05, the DEGs enrichment results in GO terms and KEGG pathways were obtained.

#### Correlation analysis

The immune system dysfunction has been suggested to play an important role in the occurrence of pulmonary tuberculosis [25]. Therefore, it is necessary to analyze the correlations of genes associated with immune system. In the present study, the Pearson correlation coefficient (PCC) was calculated across 15 samples to investigate potential regulatory relationships between important genes. The gene pairs with |PCC| > 0.5 were selected for further analysis.

#### Logistic regression analysis

In order to identify the risk biomarkers of pulmonary TB, we performed multivariate logistic regression analysis for the gene pairs with significant correlations (|PCC| > 0.5) using SPSS 19.0 software (SPSS Inc., Chicago, Illinois, USA) [26]. The genes with *p*-value < 0.05 were selected as feature genes.

## Results

### Identification of DEGs

**Fig. 1:**
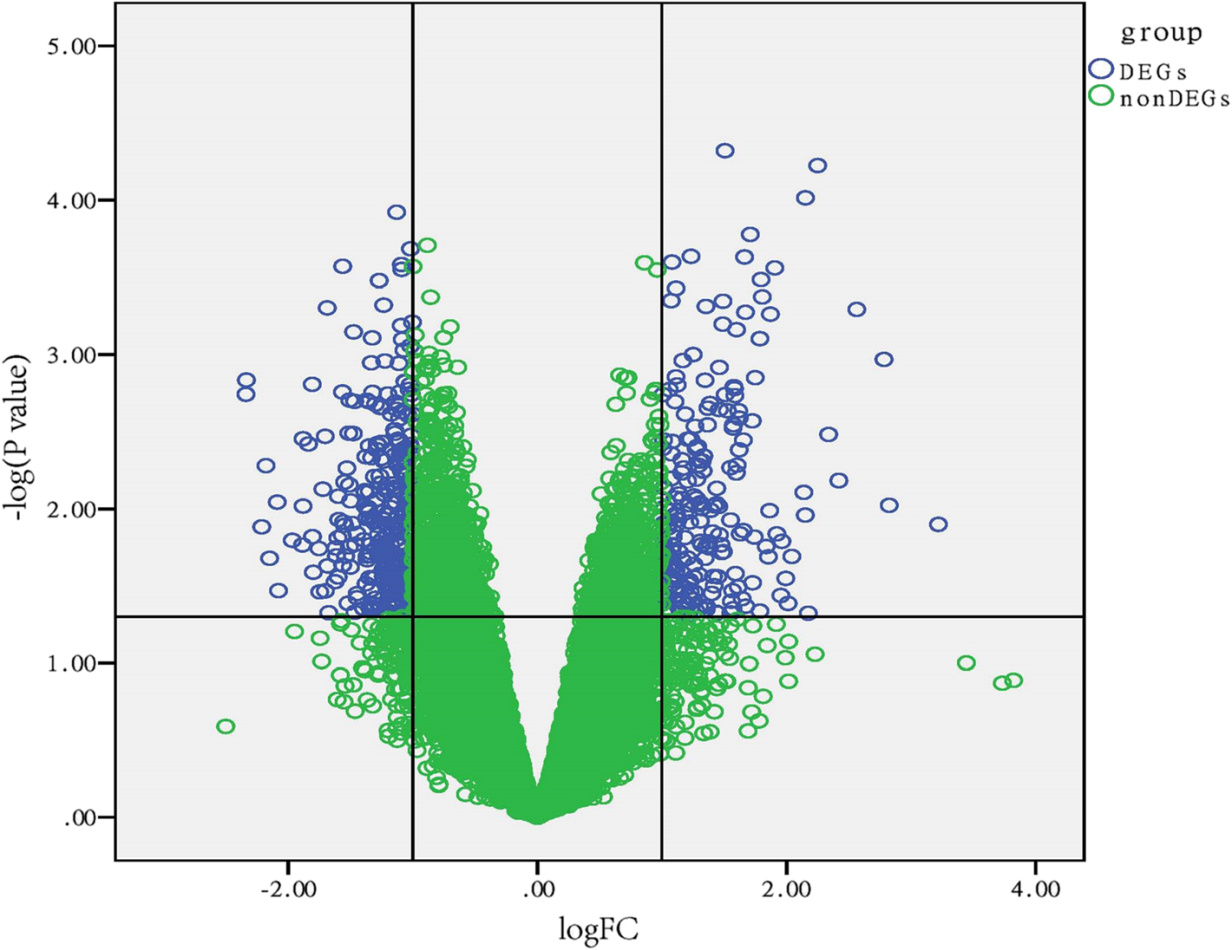
Volcano plot for the differentially expressed genes (DEGs). The x-axis represents the log_2_-fold change (log_2_FC). The y-axis represents the -log_10_ *p*-value. Blue-colored nodes are DEGs with *p*-value < 0.05 and |log_2_FC| > 1. Green-colored nodes are non-DEGs

Based on the thresholds of |log_2_FC| > 1.0 and FDR < 0.05, a total of 1084 DEGs were identified between pulmonary TB and TB latent infection samples, including 565 down-regulated genes and 519 up-regulated genes. The result was shown in volcano plot (Fig. 1).

### PPI network construction

**Fig. 2:**
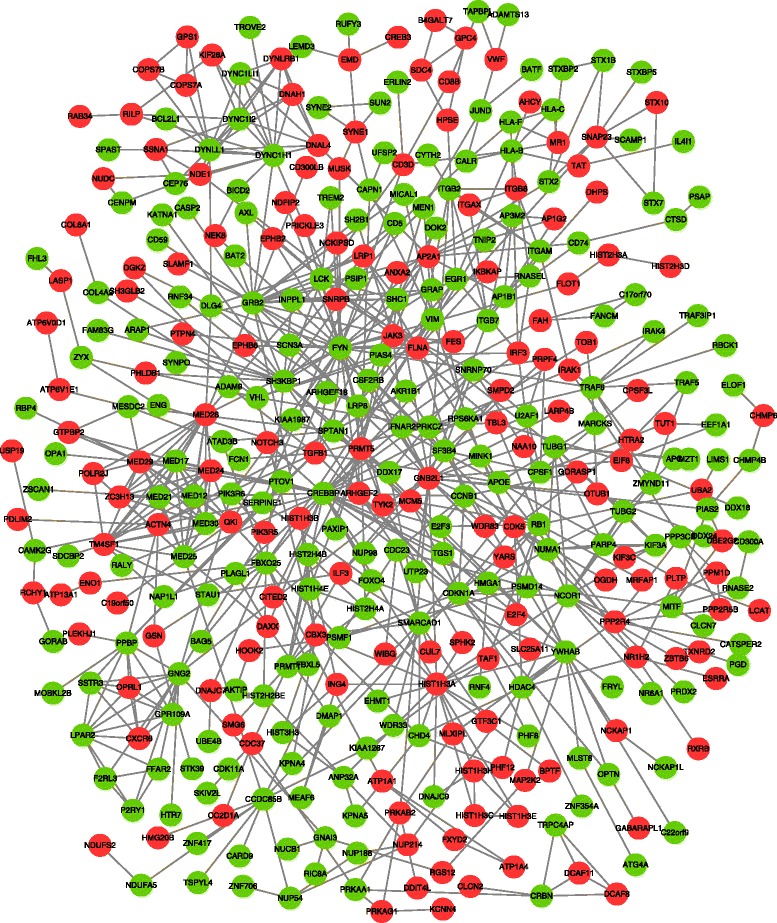
The protein-protein interaction (PPI) network of differentially expressed genes (DEGs). The green nodes stand for down-regulated genes. The red nodes stand for up-regulated genes

The PPI network consisted of 768 interaction pairs among 446 genes, including 253 down- and 193 up-regulated genes (Fig. 2). In this network, the proteins proto-oncogene tyrosine-protein kinase Fyn (*FYN*, degree = 34), CREB binding protein (*CREBBP*, degree = 28), growth factor receptor-bound protein 2 (*GRB2*, degree = 23) and guanine nucleotide binding protein (G protein) and beta polypeptide 2-like 1 (*GNB2L1*, degree = 21) were selected as hub nodes (genes) for the high connectivity degree.

### Network-based neighborhood scoring

The top 50 nodes with higher scores and the last 50 nodes with lower scores were selected and the top 5 and last 5 genes were shown in Table 1. For instance, sirtuin 5 (*SIRT5*), tyrosyl-tRNA synthetase (*YARS*), sphingomyelin phosphodiesterase 2, and neutral membrane (*SMPD2*) had scores > 0. While the scores of solute carrier family 6 (neutral amino acid transporter), member 17 (*SLC6A17*), *SCL6A8* and chloride channel, voltage-sensitive 7 (*CLCN7*) were < 0. Additionally, the down-regulated genes RIC8 guanine nucleotide exchange factor A (*RIC8A*), basic leucine zipper transcription factor, ATF-like (*BATF*) and microtubule associated monooxygenase, calponin LIM domain containing 1 (*MICAL1*), and the up-regulated genes ATPase, Na+/K+ transporting, alpha 4 polypeptide (*ATP1A4*), histone cluster 1, H3c (*HIST1H3C*), histone cluster 2, H3d (*HIST2H3D*), histone cluster 1, H3e (*HIST1H3E*) and tyrosine kinase 2 (*TYK2*) were also important genes.

**Table 1:** The top 5 gene with higher neighborhood scores and the last 5 genes with lower neighborhood scores

Gene	Neighbor score	Rank
SIRT5	1.339588	1
YARS	1.3003	2
SMPD2	1.278101	3
NAA10	1.275932	4
UQCC	1.265338	5
LOXL3	−1.36572	−5
MEN1	−1.37437	−4
CLCN7	−1.3931	−3
SLC6A8	−1.4102	−2
SLC6A17	−1.4102	−1

**Fig. 3:**
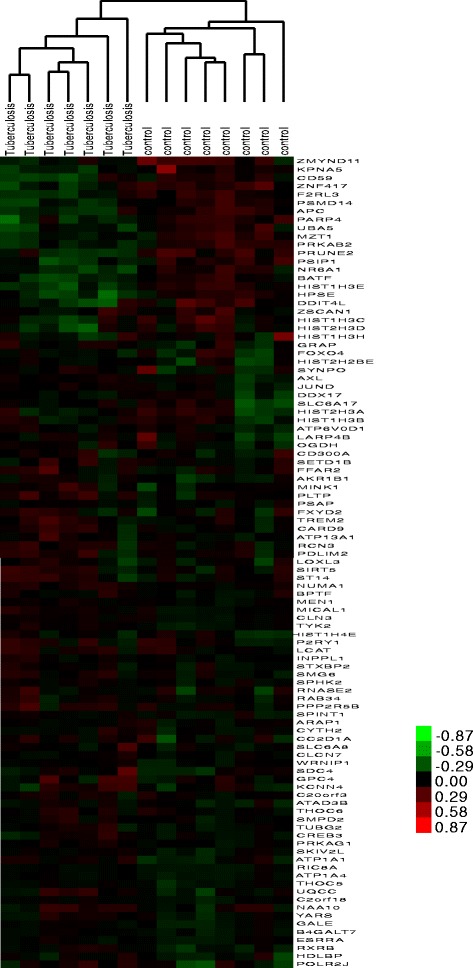
Clustering analysis of the important genes. The above dendrogram shows clustering of the samples. The red color stands for up-regulated genes, while green color stands for down-regulated genes

Furthermore, hierarchical clustering analysis for these important genes showed that these 100 important genes could differentiate the pulmonary TB samples and TB latent infection samples (Fig. 3).

### Functional enrichment analysis

GO enrichment analysis was carried out for the important genes. The significant (*p* < 0.05) GO biological process (BP) terms of up- and down-regulated genes were shown in Table 2 (*p*-values in ascending order). The down-regulated genes were significantly enriched in regulation of osteoblast differentiation (*p* = 0.00816) and positive regulation of hydrolase activity (*p* = 0.018904). Besides, the up-regulated genes were mainly related to nucleoside triphosphate biosynthetic process (*p* = 0.003512), mRNA export from nucleus (*p* = 0.004389) and purine nucleoside triphosphate metabolic process (*p* = 0.005796).

**Table 2:** The Gene Ontology (GO) biological process and the Kyoto Encyclopedia of Genes and Genomes (KEGG) pathway enrichment analyses of differentially expressed genes

Type	Category	Term	Count	*P*-value
GO				
Down-regulated genes				
GO:0045667		regulation of osteoblast differentiation	3	0.00816
GO:0051345		positive regulation of hydrolase activity	4	0.018904
GO:0030278		regulation of ossification	3	0.025314
GO:0010638		positive regulation of organelle organization	3	0.028404
GO:0045596		negative regulation of cell differentiation	4	0.030755
GO:0002076		osteoblast development	2	0.03129
GO:0045736		regulation of cyclin-dependent protein kinase activity	2	0.034366
GO:0007596		blood coagulation	3	0.041412
GO:0045667		regulation of osteoblast differentiation	3	0.00816
Up-regulated genes				
GO:0009142		nucleoside triphosphate biosynthetic process	4	0.003512
GO:0006406		mRNA export from nucleus	3	0.004389
GO:0009144		purine nucleoside triphosphate metabolic process	4	0.005796
GO:0009260		ribonucleotide biosynthetic process	4	0.006063
GO:0006405		RNA export from nucleus	3	0.006715
GO:0009150		purine ribonucleotide metabolic process	4	0.00814
GO:0006164		purine nucleotide biosynthetic process	4	0.009852
GO:0015672		monovalent inorganic cation transport	5	0.014828
GO:0006644		phospholipid metabolic process	4	0.019213
GO:0034654		nucleic acid biosynthetic process	4	0.020019
GO:0034404		nucleoside and nucleotide biosynthetic process	4	0.020019
GO:0006665		sphingolipid metabolic process	3	0.021316
GO:0046784		intronless viral mRNA export from host nucleus	2	0.023835
GO:0006812		cation transport	6	0.024009
GO:0006643		membrane lipid metabolic process	3	0.024609
GO:0051028		mRNA transport	3	0.028097
GO:0006684		sphingomyelin metabolic process	2	0.03263
GO:0050657		nucleic acid transport	3	0.034322
GO:0050658		RNA transport	3	0.034322
GO:0019216		regulation of lipid metabolic process	3	0.044558
KEGG				
up-regulated genes				
hsa04920		Adipocytokine signaling pathway	3	0.039178
hsa04260		Cardiac muscle contraction	3	0.041574

Count: enriched gene number in the GO category

In addition, 2 pathways were enriched by the up-regulated important genes (Table 2), including adipocytokine signaling pathway and cardiac muscle contraction pathway. However, the down-regulated genes were not enriched in any pathways.

### Correlation analysis

**Fig. 4:**
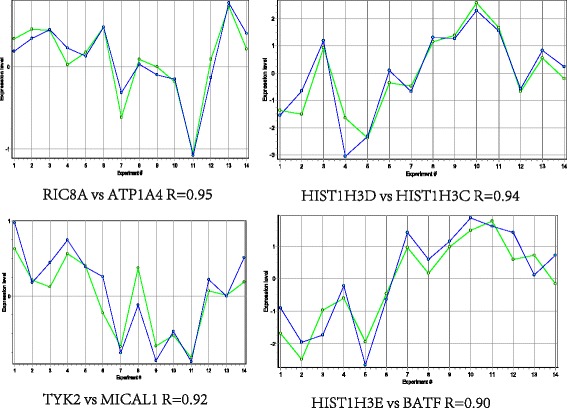
The expression levels of top 4 gene pairs. The x-coordinate represents samples; y-coordinate represents gene expression values. The blue lines represent RIC8 guanine nucleotide exchange factor A (*RIC8A*), histone cluster 2, H3d (*HIST2H3D*), tyrosine kinase 2 (*TYK2*) and histone cluster 1, H3e (*HIST1H3E*), respectively. The green lines represent ATPase, Na+/K+ transporting, alpha 4 polypeptide (*ATP1A4*), histone cluster 1, H3c (*HIST1H3C*), microtubule associated monooxygenase, calponin LIM domain containing 1 (*MICAL1*) and basic leucine zipper transcription factor, ATF-like (*BATF*), respectively. R stands for Pearson correlation coefficient

A total of 950 gene pairs were identified in Pearson correlation analysis. The top 10 highly correlated gene pairs were shown in Table 3. Specially, the expression levels of the top 4 correlated gene pairs (PCC > 0.9) were shown in Fig. 4, that was, *RIC8A*-*ATP1A4*, *HIST1H3C*-*HIST2H3D*, *HIST1H3E*-*BATF* and *MICAL1*-*TYK2*, besides, all of them showed positive correlations.

**Table 3:** The top 10 highly correlated gene pairs

Node ID1	Node ID2	Correlation
ATP1A4	RIC8A	0.95073
HIST2H3D	HIST1H3C	0.94367
HIST1H3E	BATF	0.92093
TYK2	MICAL1	0.90583
THOC5	ATP1A4	0.89967
HPSE	HIST1H3E	0.89867
F2RL3	BATF	0.8935
GALE	YARS	0.8881
PRKAB2	MZT1	0.8855
APC	PSMD14	0.86483

Correlation: Pearson correlation coefficient

### Logistic regression analysis

In order to identify the risk biomarkers of pulmonary TB, the gene pairs with significant correlations (|PCC| > 0.5) were performed logistic regression analysis. The analysis identified 80 significant feature genes, such as *ATP1A4* (*p* = 0.031), *RIC8A* (*p* = 0.035), *HIST1H3E* (*p* = 0.005), *BATF* (*p* = 0.021), *TYK2* (*p* = 0.008) and *MICAL1* (*p* = 0.011). The prediction accuracy for the two groups of samples were 100 %.

## Discussion

In this study, a total of 1084 DEGs including 565 down- and 519 up-regulated genes were selected. The up-regulated genes were mainly related to nucleoside triphosphate biosynthetic process. The down-regulated genes were significantly enriched in regulation of osteoblast differentiation. The gene pairs *RIC8A*-*ATP1A4*, *HIST1H3C*-*HIST2H3D*, *HIST1H3E*-*BATF* and *MICAL1*-*TYK2* were identified with highly positive correlations. Besides, they were selected as feature genes in logistic regression analysis.

*RIC8A* encoding protein interacts with guanine nucleotide binding protein (G protein) [27]. It has been reported that *RIC8A* controls *Drosophila* neural progenitor asymmetric division by regulating heterotrimeric G proteins [28]. G protein is an important signal transducing molecule in cells [29], which activates MAP kinase signaling [30]. Elkington et al. [31] have reported that active pulmonary TB can be mediated by MAP kinase signaling pathway. In this study, *RIC8A* was down-regulated in pulmonary TB, suggesting that *RIC8A* may be associated with the pulmonary TB development through regulating MAP kinase signaling pathway with G proteins.

ATP1A4 is a member of P-type cation transport ATPase family and belongs to Na, K-ATPase subfamily. The P-type ATPases remove Ca^2+^ against very large concentration gradients in eukaryotic cells and play an important role in intracellular calcium homeostasis [32]. Importantly, calcium homeostasis involves in apoptosis and regulates important cellular events triggered upon infection of macrophages with pathogenic mycobacteria [33]. It has been reported that the *M. tuberculosis* blocks the delivery of the Na, K-ATPases [34]. Additionally, Rao et al. [35] have shown that de novo ATP synthesis is essential for the viability of hypoxic nonreplicating mycobacteria, requiring the cytoplasmic membrane to be fully energized. Interestingly, *ATP1A4* was found enriched in nucleoside triphosphate biosynthetic process in this study. Therefore, we speculated that *ATP1A4* may play a vital role in the occurrence of pulmonary TB by controlling ATP synthesis.

*HIST1H3C*, *HIST2H3D* and *HIST1H3E* belong to histone H3 family, which are responsible for controlling the dynamics of the chromosomal fiber in eukaryotes by regulating histone acetylation. Importantly, this process is essential in modulating gene transcription through chromatin organization, and perturbation of this process can result in aberrant gene transcription and cause some diseases, including lung diseases [36, 37]. Additionally, histones play a central role in DNA repair and DNA replication [38]. Boshoff et al. [39] reported that DNA-damaging agents were rich in vivo produced by host cells due to an effort to eradicate the *M. tuberculosis*. Therefore, DNA repair-related histones may an play important role in inhibiting *M. tuberculosis* infection. In this study, the up-regulation of *HIST1H3C*, *HIST2H3D* and *HIST1H3E* may be associated with occurrence of pulmonary TB.

*BATF* belongs to the adaptor-related protein 1 (AP-1)/activating transcription factor (ATF) superfamily of transcription factors. AP-1 family transcription factors control the differentiation of lymphocyte cells in immune system [40]. Lymphocytes are crucial in the immune defence against *M. tuberculosis*, which can secrete interferons (ITFs) in response to *M. tuberculosis* infection [41]. It has been reported that ITF-γ, a product of T lymphocytes, contributes to protective immunity against *M. tuberculosis* by activating macrophages in pulmonary TB [42]. Taken together, although the role of *BATF* in pulmonary TB has not been studied, we speculate that *BATF* may be involved in the occurrence of TB via immune system.

*TYK2* encodes a tyrosine kinase belonging to Janus kinases (JAKs) family. It has been reported that JAKs are activated following interactions between cytokines and their cognate receptors on cell surface [43]. *TYK2* negatively regulates adaptive Th1 immunity by mediating IL-10 signaling and promoting ITF-γ-dependent IL-10 reactivation [43]. Redford et al. [44] have reported that IL-10 can suppress the functions of macrophage and dendritic cell, which were required for the capture, control and initiation of immune responses to *M. tuberculosis*. Therefore, *TYK2* may involve in the pathogenesis of pulmonary TB via regulating *IL-10*. For MICAL1, it can act as a cytoskeletal regulator [45]. Specially, cell migration and phagocytosis are critically dependent on cytoskeletal rearrangements [46]. It has been reported that cell migration and phagocytosis are important for resistance against pulmonary TB [47]. Therefore, the down-regulation of *MICAL1* may be related to pulmonary TB via controlling cell migration and phagocytosis.

## Conclusions

In conclusion, the present study identified several key gene pairs (*RIC8A*-*ATP1A4*, *HIST1H3C*-*HIST2H3D*, *HIST1H3E*-*BATF*, *MICAL1*-*TYK2*) associated with pulmonary TB or TB latent infection by comprehensive bioinformatics methods, which may provide new insights for the diagnosis and treatment of this disease. However, this study had some limitations. On the one hand, the sample size was small which might cause a high rate of false positive results. Secondly, there was no experimental verification. Therefore, further genetic and experimental studies with larger sample sizes are still needed to confirm the findings in this study.

## Abbreviations

AP-1: Adaptor-related protein 1
ATF: Activating transcription factor
*ATP1A4*: ATPase, Na+/K+ transporting, alpha 4 polypeptide
*BATF*: Basic leucine zipper transcription factor, ATF-like
BH: Benjamin and Hochberg
BP: Biological process
*CLCN7*: Chloride channel, voltage-sensitive 7
*CREBBP*: CREB binding protein
DAVID: Database for annotation, visualization and integrated discovery
DEGs: Differentially expressed genes
*FYN*: Tyrosine-protein kinase Fyn
G protein: Guanine nucleotide binding protein
*GNB2L1*: Guanine nucleotide binding protein and beta polypeptide 2-like 1
GO: Gene Ontology
*GRB2*: Growth factor receptor-bound protein 2
*HIST1H3C*: Histone cluster 1, H3c
*HIST1H3E*: Histone cluster 1, H3e
*HIST2H3D*: Histone cluster 2, H3d
HPRD: Human protein reference database
ITFs: Interferons
JAKs: Janus kinases
KEGG: Kyoto Encyclopedia of Genes and Genomes
*MICAL1*: Microtubule associated monooxygenase, calponin LIM domain containing 1
PCC: Pearson correlation coefficient
PPI: Protein-protein interaction
*RIC8A*: RIC8 guanine nucleotide exchange factor A
*SIRT5*: Sirtuin 5
SLC6A17: Solute carrier family 6neutral amino acid transporter), member 17
*SMPD2*: Sphingomyelin phosphodiesterase 2, and neutral membrane
STRING: search tool for the retrieval of interacting genes
TB: Pulmonary tuberculosis
*TYK2*: Tyrosine kinase 2
*YARS*: Tyrosyl-tRNA synthetase
